# The dynamic change of serotype distribution and antimicrobial resistance of pneumococcal isolates since PCV13 administration and COVID-19 control in Urumqi, China

**DOI:** 10.3389/fcimb.2023.1110652

**Published:** 2023-01-31

**Authors:** Meng-Yang Guo, Xing-Hai Shi, Wei Gao, Ju-Ling Tian, Lin Yuan, Juan Yang, Dilinuer Wumaier, Jiang Cao, Reziwaguli Abulimiti, Wen-Li Zhang, Kai-Hu Yao

**Affiliations:** ^1^ Ministry of Education (MOE) Key Laboratory of Major Diseases in Children, Beijing Key Laboratory of Pediatric Respiratory Infection Diseases, Beijing Pediatric Research Institute, Beijing Children’s Hospital, Capital Medical University, National Center for Children’s Health, Beijing, China; ^2^ Medical Laboratory, Urumqi Children’s Hospital, Beijing, China

**Keywords:** *Streptococcus pneumoniae*, serotype, drug resistance, children, COVID-19

## Abstract

**Objective:**

This study aims to analyze the serotype distribution and drug resistance of *Streptococcus pneumoniae* isolated from children aged 8 days to 7 years in Urumqi, China, between 2014 to 2021, during which PCV13 was introduced in the private sector’s immunization program and COVID-19 control was administrated in the last 2 years.

**Methods:**

Serotypes of *S. pneumoniae* isolates were determined by Quellung reaction, and their susceptibility against 14 antimicrobials were tested. According to the start year of PCV13 administration (2017) and COVID-19 control (2020), the study period was divided into three stages: 2014–2015, 2018–2019, and 2020–2021.

**Results:**

A total of 317 isolates were involved in this study. The most common serotypes were type 19F (34.4%), followed by 19A (15.8%), 23F (11.7%), 6B (11.4%), and 6A(5.0%). The coverage rate of both PCV13 and PCV15 was 83.0%. The coverage of PCV20 was a little higher at 85.2%. The resistance rate against penicillin was 28.6% according to the breakpoints of oral penicillin, which would reach up to 91.8% based on the breakpoints of parenteral penicillin for meningitis. The resistance rates to erythromycin, clindamycin, tetracycline, and sulfamethoxazole-trimethoprim were 95.9%, 90.2%, 88.9%, and 78.8%, respectively. The PCV13 isolate was more resistant to penicillin than the non-PCV13 ones. There was not any significant change found in the serotype distribution since the PCV13 introduction and the COVID-19 control. The resistance rate against oral penicillin slightly elevated to 34.5% in 2018–2019 from 30.7% in 2014–2015 and then decreased significantly to 18.1% in 2020–2021 (*χ* ^2^ = 7.716, *P* < 0.05), while the resistance rate to ceftriaxone (non-meningitis) continuously declined from 16.0% in 2014–2015 to 1.4% in 2018–2019 and 0% in 2020–2021 (Fisher = 24.463, *P* < 0.01).

**Conclusion:**

The common serotypes of *S. pneumoniae* isolated from children in Urumqi were types 19F, 19A, 23F, 6B, and 6A, which we found to have no marked change since the PCV13 introduction and the COVID-19 control However, the resistance rate to oral penicillin and ceftriaxone significantly declined in the COVID-19 control stage.

## 1 Introduction

*Streptococcus pneumoniae* (*S. pneumoniae*) is the main pathogen causing pneumonia, meningitis, bacteremia, and other serious diseases in children. It is also a common cause of acute otitis media and sinusitis. It is an important cause of morbidity and death of infants in China. The number of pneumococcal disease cases in children under 5 years old in China accounts for 12% of the total number of cases in the world, thus ranking as the second country in the world with the highest occurrence rate (Chinese Preventive Medicine Association and Vaccine and Immunology Branch of the Chinese Preventive Medicine Association, 2020). Universal immunization with pneumococcal conjugate vaccines (PCVs) in children can effectively prevent invasive and some non-invasive pneumococcal infections (Moore et al., 2015). Therefore, the World Health Organization recommends that countries should include PCVs in children’s immunization program (World Health Organization, 2019). A systematic study on the epidemiology of pneumococci in children in mainland of China from 2000 to 2018 showed that the 13-valent PCV (PCV13, including 1, 3, 4, 5, 6A, 6B, 7F, 9V, 14, 18C, 19F, 19A, and 23F serotypes) coverage was 90.4%, which suggested the potential effect of this vaccination (Men et al., 2020). Another systematic review including data from 2006 to 2016 found no significant difference in the coverage of PCV13 in invasive isolates compared to that in non-invasive strains (Lyu et al., 2017). However, the previous domestic investigations on the serotypes of *S. pneumoniae* isolates in children were mainly performed in developed areas and cities, which were mainly settled by the Han people. Similar investigations in underdeveloped areas or on ethnic minorities, especially of the population in the northwest, were rare. PCV13 was available for private choice in China in November 2016. It has not been widely administrated, especially in undeveloped areas, because of its high price. Since the beginning of 2020, COVID-19 controls were globally preformed, which have been implemented until now in China. The change in the epidemiology of *S. penumonaie* following the epidemic of COVID-19 has attracted the attention of researchers. Sempere et al. reported that the use of antimicrobials to prevent co-infections in patients with COVID-19 might have affected the increased proportion of pneumococcal-resistant strains (Sempere et al., 2022).

In the present study, the serotypes and drug resistance of *S. pneumoniae* isolated from children in Urumqi Children’s Hospital from 2014 to 2021 were analyzed, which would add new knowledge on the epidemiology of *S. pneumoniae* in China and on the effect of PCV administration and COVID-19 control.

## 2 Methods

### 2.1 Bacterial isolates and study period

*S. pneumoniae* isolates identified and frozen at -80°C during daily work at the Medical Laboratory Department of the Children’s Hospital in Xinjiang Urumqi from 2014 to 2021 were recovered and re-identified by optochin test, bile solubility test, and capsule swelling test (Satzke et al., 2013). The isolates should be cultured from various children. According to the start year of PCV13 administration (2017) and COVID-19 control (2020), the study period was divided into three stages: 2014–2015, 2018–2019, and 2020–2021.

This retrospective study did not record the information that can confirm the identity of the children or their guardians. Informed consent was not required, and this was approved by the hospital ethics committee (2017-142).

### 2.2 Serotyping

Antisera (Statens Serum Institute, Copenhagen, Denmark) were used to determine the serotype of the isolates by capsular swelling test, that is, Quellung test (Satzke et al., 2013). The constituent ratios of each serotype were calculated, and the coverage rate of *S. pneumoniae* vaccines was estimated by the sum of the serotype constituent ratio of vaccine serotypes.

### 2.3 Antimicrobial susceptibility testing

The minimal inhibitory concentration (MIC) of penicillin, amoxicillin, cefotaxime, and ceftriaxone was detected with E-test stripes, and the susceptibility of meropenem, erythromycin, sulfamethoxazole-trimethoprim (SMZ-TMP), tetracycline, chloramphenicol, linezolid, vancomycin, levofloxacin, and moxifloxacin was detected by VITEC 2 Compact System (bioMerieux Inc., NC, USA). The susceptibility of clindamycin was evaluated by disk diffusion test (Oxoid Ltd., Basingstoke, UK). The quality control strain for this test was *S. pneumoniae* ATCC49619. The susceptibility was judged according to the breakpoints recommended by the Clinical and Laboratory Standards Institute (Clinical and Laboratory Standards Institute (CLSI), 2019).

### 2.4 Statistical analysis

A database including the age, nationality, and sex of the patients and the serotype and drug resistance of the corresponding isolate was constructed in Microsoft Excel 2003. The susceptibility rate, intermediary rate, and drug resistance rate of different antimicrobials were calculated. SPSS25.0 (IBM SPSS Inc., Chicago, IL, USA) was used for statistical analysis. The significance of each group was compared by *χ* ^2^ test and Fisher’s exact test. *P <*0.05 was considered to be statistically significant.

## 3 Results

### 3.1 Characteristics of strains and specimens and demography

A total of 317 strains were successfully recovered and were tested in the present study, which included 75 strains isolated in 2014 to 2015, 148 strains in 2018 to 2019, and 94 strains in 2020 to 2021. Among all strains, 298 were isolated from sputum (94.0%). A few strains were cultured from eye secretions (3.5%), ear secretions (1.3%), blood (0.9%), and bronchoalveolar lavage fluid (0.3%).

The age composition of host children ranged from 8 days to 7 years old, which included 188 cases of <2 years old (59.3%), 113 cases of 2–< 5 years old (35.6%), and 16 cases of 5–7 years old (5.0%). There were 194 male children (61.9%) and 123 female children (38.8%). Among the 317 cases, there were 202 cases from Han people (63.7%) and 115 cases from ethnic minority people (36.3%), including 92 cases from Uygur, 17 cases from Kazak, four cases from Mongolian, and two cases from Hui.

### 3.2 Serotype distribution and pneumococcal vaccine coverage

**Figure 1:**
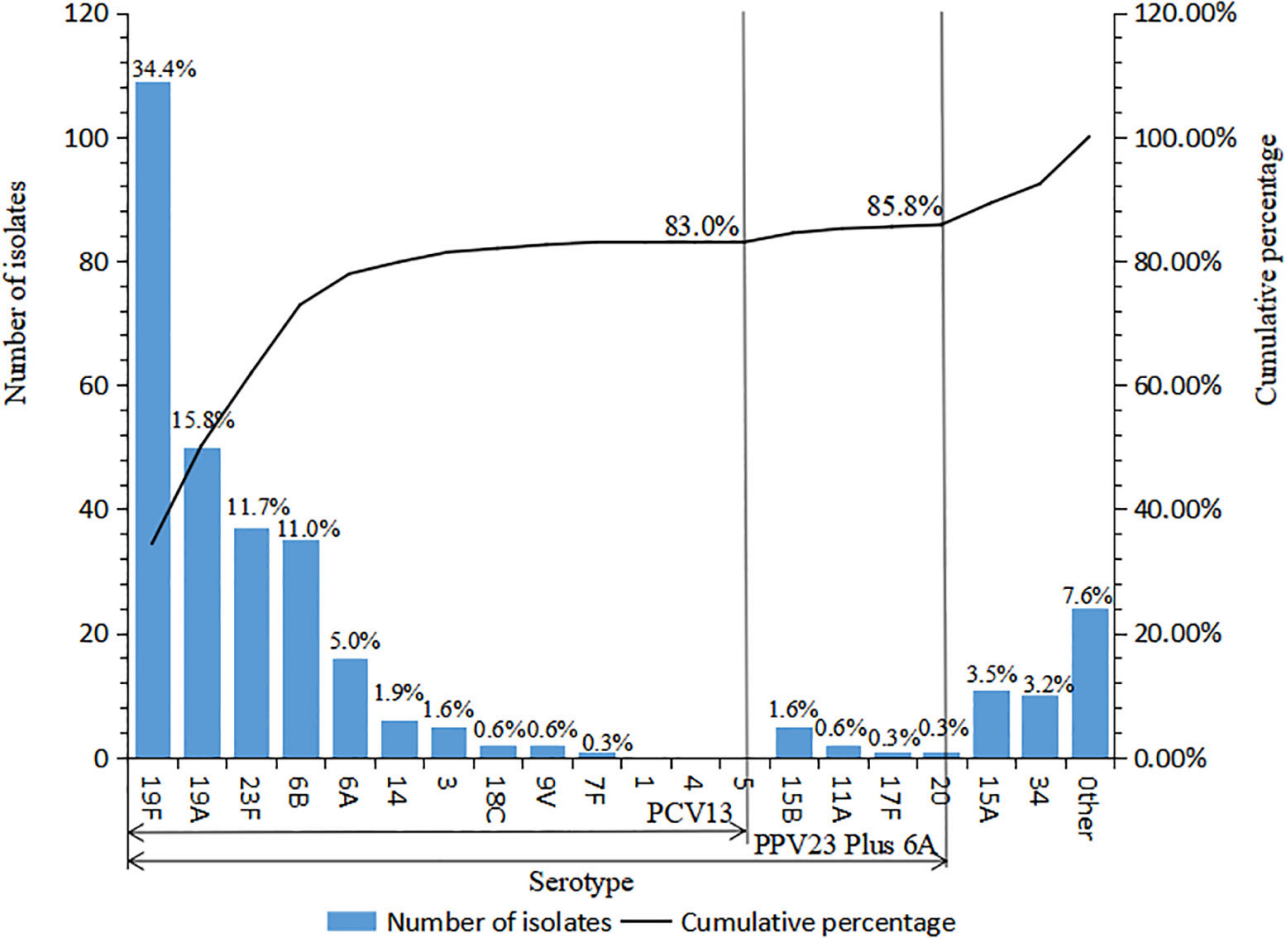
Serotype distribution of 317 *S. pneumoniae* strains isolated in Urumqi Children’s Hospital during 2014-2021" .The note of Figure 1 is "Other serotypes included 4 strains of type 6C, 3 strains of each type 23A, 7C, 16F, 2 strains of type 42, and 1 strain of each type 29, 13, 37, 23B, 21, 15C, 10C, 28A, and 15F".

A total of 30 serotypes were identified in the present 317 strains. The serotype distribution and the cumulative coverage of PCV13 and 23-valent capsular polysaccharide vaccine (PPV23, covering serotypes 1, 2, 3, 4, 5, 6B, 7F, 8, 9N, 9V, 10A, 11A, 12F, 14,15B, 17F, 18C, 19A, 19F, 20, 22F, 23F, and 33F) are shown in **Figure 1**. The most common serotype was 19F (109 strains, 34.4%), followed by 19A (50, 15.8%), 23F (37, 11.7%), 6B (35, 11.4%), 6A (16, 5.0%), 15A (11, 3.5%), and 34 (10, 3.2%). The coverage rate of PCV13 reached 83.0% (263/317). The additional types 22F and 33F in PCV15 were not identified in the study. The coverage of PCV20 increased slightly to 85.2% (270/317), in which the further additional types 8, 10A, and 12F based on PCV15 were not found either. The coverage rate of PPV23 was 80.8% (256/317).

A recent and newly reported PCV, V116 (containing types 3, 6A, 7F, 8,9N, 10A, 11A, 12F, 15A, 16F, 17F, 19A, 20, 22F, 23A, 23B, 24F, 31, 33F, 35B, and de-O-acetylated 15B) (Platt et al., 2022), covered 30.0% (95/317) of the present isolates, 22.7% (72/317) of which were the same serotypes with PCV13 (types 3, 6A, 7F, and 19A), and the other 7.3% (23/317) were unique types to V116 (types 11A, 15A, 17F, 20, 23A, 23B, and de-O-acetylated 15B).

**Figure 2:**
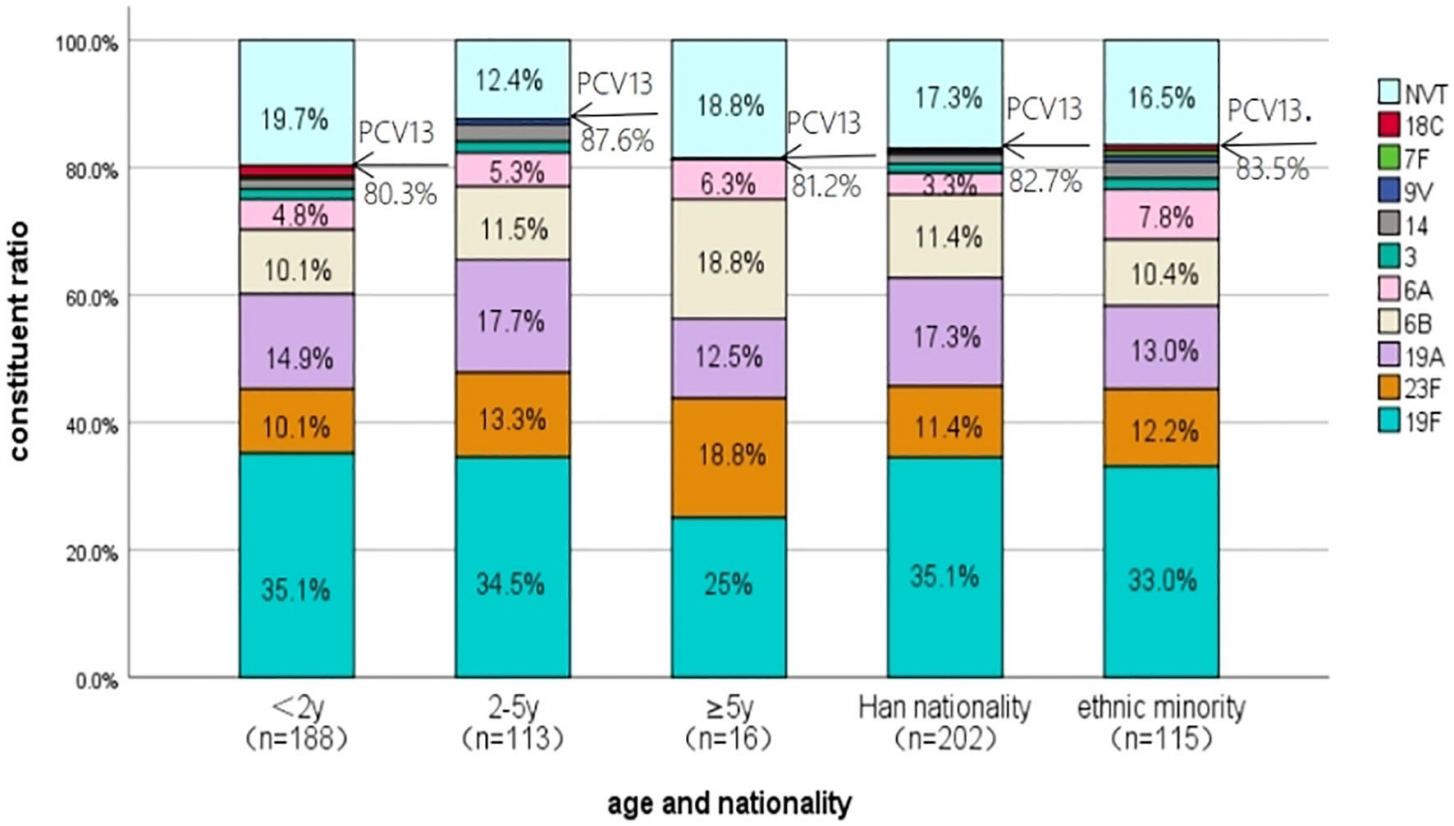
The serotype distribution of *S. pneumoniae* isolated from children in different age groups and different ethnic peoples in Urumqi Children’s Hospital during 2014-2021". The note of Figure 2 is " NVT represents the serotypes not covered by the PCV13.

The comparison of serotype composition and PCV13 coverage by age groups and different ethnic peoples is shown in **Figure 2**. The type distributions of the isolates cultured from children <2 years old, 2–5 years old, and ≥5 years old were similar, and the PCV13 coverage rates were also comparable with each other (80.3% *vs*. 87.6% *vs*. 81.2%, *χ* ^2^ = 2.690, *P* > 0.05). No significant difference in serotype distribution was found between Han children and ethnic minority children as well as in terms of the PCV13 coverage rates (82.7% *vs*. 83.5%, *χ* ^2^ = 0.034, *P* > 0.05).

The serotype distribution of the strains and the coverage of PCV13 in different study stages are shown in **Table 1**. The coverage rate of PCV13 in 2018–2019 and 2020–2021, respectively, was lower than the corresponding data in 2014–2015, although these differences were not statistically significant. For common serotypes, the proportion of serotype 19A decreased significantly since the PCV13 administration, and that of type 23F was found obviously fluctuated in the three stages. The rates of non-PCV13 types increased from 8.0% in 2014–2015 to 20.3% in 2018–2019 and to 19.1% in 2020–2021. However, this increase did not reach statistical significance (*χ* ^2^ = 5.725, *P* > 0.05).

**Table 1:** Distribution of serotypes of *S. pneumoniae* strains isolated from different year periods.

Serotype	2014–2015 (*n* = 75)	2018–2019 (*n* = 148)	2020–2021 (*n* = 94)	*χ* ^2^	*P*
PCV13	69 (92.0%)	118 (79.7%)	76 (80.9%)	5.725	0.057
19F	25 (33.3%)	53 (35.8%)	31 (33.0%)	0.253	0.881
19A	17 (22.7%)	25 (16.9%)	8 (8.5%)	6.554	0.038
6B	8 (10.7%)	14 (9.5%)	13 (13.8%)	1.132	0.568
23F	13 (17.3%)	10 (6.8)	14 (14.9%)	6.746	0.034
6A	4 (5.3%)	9 (6.1%)	3 (3.2%)	1.018	0.601
Others^a^	2 (2.7%)^b^	7 (4.7%)^c^	7 (7.4%)^d^	2.047	0.359
Non-PCV13	6 (8.0%)	30 (20.3%)	18 (19.1%)	5.725	0.057

a Other serotypes in PCV13 except 19F, 19A, 6B, 23F, and 6A.

b Others include one serotype 9V strain and one serotype 14 strain in 2014–2015.

c Others include four strains of serotype 3, two strains of serotype 14, and one strain of serotype 18C in 2018–2019.

d Others include three strains of serotype 14 and one strain of serotype 3, 9V, 18C, and 7F in 2020-2021.

### 3.3 Antimicrobial susceptibility

The susceptibility of the 317 strains of *S. pneumoniae* to 14 antimicrobials is shown in **Table 2**. All strains were susceptible to vancomycin and moxifloxacin; almost all were also susceptible to levofloxacin and linezolid (99.7% and 99.3%, respectively). The susceptibility rate to erythromycin, clindamycin, SMZ-TMP, and tetracycline was very low and less than 12%. The susceptibility to penicillin, ceftriaxone, and cefotaxime appeared to have been divided based on the breakpoints—for example, when the breakpoints of parenteral penicillin (non-meningitis) were adopted (susceptible ≤2 mg/L, intermediate = 4 mg/L, and resistant ≥8 mg/L), the susceptibility rate could reach 97.5%. However, the susceptibility rates based on the parenteral penicillin (meningitis) breakpoints (susceptible ≤0.06 mg/L and drug resistant ≥0.12 mg/L) or the oral penicillin points (susceptible ≤0.06 mg/L, intermediate 0.12–1 mg/L, and drug resistant ≥2 mg/L) were only 8.2% and 7.9%.

**Table 2:** Antimicrobial susceptibility test results of 317 isolates of *S. pneumoniae* isolated in Urumqi, 2014–2021.

Antimicrobials	Number	Susceptibility			MIC (mg/L)		
		S (%)	I (%)	R (%)	MIC50	MIC90	MIC range
Penicillin	317				1	2	≤0.06–≥16
Oral	317	7.9	63.5	28.6			
Parenteral, non-meningitis	317	97.5	1.3	1.3			
Parenteral, meningitis	317	8.2	–	91.8			
Ceftriaxone	317				1	1	≤0.06–≥4
Non-meningitis	317	91.8	3.8	4.4			
Meningitis	317	40.1	51.7	8.2			
Cefotaxime	317				1	2	≤0.06–≥4
Non-meningitis	317	87.4	6.6	6			
Meningitis	317	29	58.4	12.6			
Erythromycin	315	3.2	0.9	95.9	≥ 1	≥ 1	≤0.25–>8
Clindamycin	317	8.8	1.0	90.2	–	–	–
Sulfamethoxazole-trimethoprim	292	11.3	9.9	78.8	8	≥ 16	≤0.25–≥16
Tetracycline	316	9.8	1.3	88.9	≥ 16	≥ 16	≤1–>16
Chloramphenicol	292	92.1	–	7.9	≤ 2	4	≤0.06–≥32
Levofloxacin	317	99.7	0.3	0	≤ 0.5	1	≤0.06–4
Vancomycin	317	100	–	–	≤ 1	≤ 1	≤0.5–8
Moxifloxacin	317	100%	0	0	≤ 0.25	0.25	≤0.25–1
Amoxicillin	317	89.6	7.3	3.1	1	3	≤0.06–≥8
Meropenem	312	79.5	15.4	5.1	0.25	0.5	≤0.06–2
Linezolid	273	99.3	–	–	≤ 2	≤ 2	≤1–≥16

S, susceptible; I, intermediate; R, resistant; -, no such interpretation standards.

The comparison of the susceptibility of different serotypes to penicillin and ceftriaxone is shown in **Tables 3**, **4**. The isolates covered by PCV13 showed higher I% and/or R% or MIC90 or maximum MICs to penicillin and ceftriaxone than the non-PCV13 types. According to the MICs and intermediate and resistance rates on oral penicillin breakpoints, the common serotypes covered by PCV13, including types 19F, 19A, 6B, 23F, and 6A, showed higher resistance than those of uncommon PCV13 types and non-PCV13 types. Overall, a similar trend could be found in the resistance to ceftriaxone according to the MICs and meningitis breakpoints.

**Table 3:** Susceptibility of *S. pneumoniae* to penicillin according to serotype.

Serotype	Number	MIC50(mg/L)	MIC90 (mg/L)	MIC range (mg/L)	Oral penicillin		Parenteral penicillin		
							Meningitis	Non-meningitis	
					I (%)	R (%)	R (%)	I (%)	R (%)
PCV13	263	1	2	≤0.06–≥16	63.1	33.5	96.2	1.5	1.5
19F	109	1	2	0.12–≥16	60.6	39.4	100.0	3.7	1.8
19A	50	1	≥2	≤0.06–8	48.0	50.0	98.0	0	2.0
6B	35	1	2	≤0.06–2	80.0	17.1	97.1	0	0
23F	37	1	2	≤0.06–2	73.0	24.3	97.3	0	0
6A	16	0.75	2	0.12–2	87.5	12.5	100.0	0	0
3	5	≤0.06	≤0.5	≤0.06–≤0.5	40.0	0	20.0	0	0
14	6	1	8	1–8	50.0	50.0	100.0	0	16.7
Other^a^	5	≤0.06	0.12	≤0.06–0.12	40.0	0	40.0	0	0
Non-PCV13	54	0.25	1	≤0.06–2	64.8	5.6	70.4	0	0
15A	11	1	1	≤0.06–2	72.7	9.1	81.8	0	0
15B	5	0.5	1	≤0.06–1	80.0	0	80.0	0	0
34	10	0.25	0.25	≤0.06–0.5	80.0	0	80.0	0	0
Total	317	1	2	≤0.06–16	63.5%	28.6	91.8	1.3	1.3

I, intermediate; R, resistant.

a Other includes two isolates for type 9V, two isolates for type 18C, and one isolate for type 7F which is covered by PCV13.

**Table 4:** Comparison of the susceptibility of *S. pneumoniae* to ceftriaxone between different serotypes.

Serotype	Number	MIC50 (mg/L)	MIC90 (mg/L)	MIC range (mg/L)	Meningitis		Non-meningitis	
					I (%)	R (%)	I (%)	R (%)
PCV13	263	1	1	≤0.06–≥4	56.7	9.9	4.6	5.3
19F	109	1	≥ 4	0.12–≥4	58.7	18.3	5.5	12.8
19A	50	1	1	≤0.06–2	64.0	10.0	10.0	0
6B	35	1	1	≤0.06–1	62.9	0	0	0
23F	37	1	1	≤0.06–2	48.6	2.7	2.7	0
6A	16	0.5	1	≤0.06–1	43.8	0	0	0
3	5	≤ 0.06	0.5	≤0.06–0.5	0	0	0	0
14	6	1	1	1–1	100.0	0	0	0
Other^a^	5	≤ 0.06	0.12	≤0.06–0.12	0	0	0	0
Non-PCV13	54	0.25	1	≤0.06–1	27.8	0	0	0
15A	11	1	1	≤0.06–1	63.6	0	0	0
15B	5	0.5	1	≤0.06–1	40.0	0	0	0
34	10	0.12	0.25	≤0.06–0.25	0	0	0	0
Total	317	1	1	≤0.06–≥4	51.7	8.2	3.8	4.4

I, intermediate; R, resistant.

a Other includes two isolates for type 9V, two isolates for type 18C, and one isolate for type 7F which is covered by PCV13.

The intermediate, resistance, and non-susceptibility rates of the present strains to oral penicillin and ceftriaxone (non-meningitis), meropenem, and tetracycline are found to be significantly different between various stages, as shown in detail in **Table 5**. No other statistically significant difference is achieved when drug susceptibility is compared between different years.

**Table 5:** Comparison of the susceptibility of *S. pneumoniae* to penicillin (oral) and ceftriaxone (non-meningitis) between different years.

Antimicrobials	2014–2015 (*n* = 75)	2018–2019 (*n* = 148)	2020–2021 (*n* = 94)	*χ* ^2^/Fisher	*P*
Penicillin (oral)^a^					
I (%)	62.7	56.8	74.5	7.795	0.020
R (%)	30.7	34.5	18.1	7.716	0.021
NS (%)	93.3	91.2	92.6	0.343	0.843
Ceftriaxone (non-meningitis)^a^					
I (%)	6.7	4.1	1.1	3.651	0.156
R (%)	16.0	1.4	0	24.463	<0.01
NS (%)	22.7	5.4	1.1	24.924	<0.01
Meropenem^a^					
I (%)	61.3	0	2.1	142.546	<0.01
R (%)	14.7	2.1	2.1	18.464	<0.01
NS (%)	76.0	2.1	4.3	186.598	<0.01
Tetracycline					
I (%)	0	2.0	1.1	1.240	0.814
R (%)	97.3	85.0	88.3	7.681	0.021
NS (%)	97.3	87.1	89.4	6.011	0.052

I, intermediate; R, resistant.

## 4 Discussion

The present results revealed that the common serotypes of *S. pneumoniae* isolated from children in Urumqi were types 19F (34.4%), 19A (15.8%), 23F (11.7%), 6B (11.4%), and 6A (5.0%), which showed some difference against previous data in China. The previous systematic review showed that the common serotypes of *S. pneumoniae* in Chinese children from 2006 to 2016 were types 19F, 19A, 23F, 14, and 6B (Lyu et al., 2017). In this study, serotype 14 is rarely found. It may be associated with the fact that almost all the present strains were isolated from non-invasive respiratory tract samples. The systematic review has reported that serotype 14 could be determined more frequently in the invasive strains (5.6%–35.9%) than in the non-invasive ones (0%–12.7%) (Lyu et al., 2017).

The coverage rate of PCV13 in this investigation was 83.0%, which was about the middle level as the previous surveys in Chinese children for non-invasive strains (59.0%–95.2%) and for invasive strains (76.2%–98.8%) (Lyu et al., 2017). The high coverage suggested the potential preventive effect of this vaccination for these children in this region. To date, PCV13 is only available in the private sector and inoculated infrequently. One investigation showed that less than 17% of eligible Chinese children could be immunized with PCVs (Wang et al., 2021). This study revealed that the coverage rates of PCV13 decreased from 92.0% in 2014–2015 to 79.7% in 2018–2019 and 80.9% in 2020–2021, which was hard to pinpoint with certainty as an effect of vaccination. Except for serotype 19A, most of the PCV13 types were not found to be decreased since the PCV13 administration. It was noted that the proportion of non-PCV13 types increased without statistical significance. After routine immunization with PCVs abroad, the proportion of invasive pneumococcal disease in children caused by some non-vaccine types (NVTs) was found to have increased in many surveys, and serotypes 33F, 22F, 12F, 15B, 15C, 23A, 24F, 23B, 10A, and 38 were the common NVTs (Balsells et al., 2017). The epidemic of non-PCV13 serotypes could counteract the benefits of universal immunization and require additional formula on the PCV13. However, the coverages of PCV15 or PCV20 did not reach up to higher levels significantly in this analysis. In the present study, the non-PCV13 serotypes were very dispersed and included 20 types. Among the above-mentioned common NVTs after PCV13 immunization, only types 15B, 15C, 23A, and 23B were found, which respectively accounted for only 1.6%, 0.3%, 0.9%, and 0.3% in all isolates. These findings make it very important to monitor the serotype distribution in the future.

The previous study revealed obvious differences of serotype distribution between the southern regions and the northern regions of China (Lyu et al., 2017). However, it is not clear whether these differences were associated with the ethnic composition. The present results showed that there was no difference in serotype distribution between children from different ethnic peoples. This finding suggested that the difference of serotype distribution of *S. pneumoniae* between different regions was not related to the composition of ethnic peoples. This means that it is more important to involve subjects from many sites for the investigations than to recruit subjects from different ethnic peoples.

The limited administration of PCV13 and the inconsistent epidemiological changes of PCV13 serotypes 19A and 23F in the three temporal stages complicated the interpretation for the changes of serotype distribution in this study. Considering that the serotypes were proved to be associated with other bacterial characteristics, including virulence and antimicrobial susceptibility (Zhou et al., 2022), these changes of types might be related to multiple factors, including the disease and the severity of inpatients and the increasing improvement and implementation of the antibiotic management system in recent years, especially during COVID-19 control. After the epidemic of COVID-19, various parts of China have introduced measures to control the individual purchasing of antibiotics, such as not to buy antimicrobials at drugstores or the buyer must have a nucleic acid test report. At the same time, more rules were made to control antibiotic prescription in hospitals. These measures may lead to a reduction of the spreading of drug-resistant isolates. According to the China Antimicrobial Surveillance Network, the proportion of penicillin-insensitive and penicillin-resistant strains of non-meningococcal *S. pneumoniae* in Chinese children before COVID-19 control was higher than that after COVID-19 control, and the detection rate of MRSA and MRSE in 2020–2021 was also lower than that before COVID-19 control (China antimicrobial surveillance network(CHINET), 2021). In this study, the drug resistance to ceftriaxone decreased significantly with stages, which may reflect the strict management of broad-spectrum antimicrobials and reduce the advantage of the spread of highly drug-resistant strains. However, the above-mentioned hypothesis may not be the complete interpretation for the present results. Other COVID-19 control measures, such as wearing a mask and keeping social distance, could also play a role to limit the spread of pathogens, especially in the hospital. Since COVID-19 occurred, the nosocomial infection control measures had been strengthened as never before. These measures could influence the spread of drug-resistant pathogens.

The penicillin susceptibility of *S. pneumoniae* was analyzed to get different distributions according to breakpoints for administration route and type of disease, which could be 91.8% based on the parenteral administration and meningitis breakpoints. The same was true for susceptibility to other cephalosporins. The beta-lactam antimicrobials were often recommended as the initial empirical treatment for children with *S. pneumoniae* meningitis (van de Beek et al., 2016; China National Clinical Research Center for Respiratory Diseases et al., 2020). However, the present results suggested that these drugs should not have been the initial choice if *S. pneumoniae* meningitis was suspected in Urumuqi. For the patient infected with low susceptibility or resistance to cephalosporins, vancomycin combined with cefotaxime or ceftriaxone should be prescribed. If the child is allergic to beta-lactam antimicrobials, vancomycin combined with rifampicin can be used for empirical treatment (van de Beek et al., 2016; China National Clinical Research Center for Respiratory Diseases et al., 2020). Similar to a previous report (Lyu et al., 2017), this study also found that the isolates covered by PCV13 were more non-susceptible to antimicrobials than the NVT isolates. This suggests that PCV13 immunization would not only prevent pneumococcal infections but also control the spread of drug resistance of *S. pneumoniae* (Ben-Shimol et al., 2020).

There are several limitations in the present study. First, the isolates frozen in 2016 to 2017 could be recovered for some technological reasons. The natural fluctuation of serotype distribution and drug resistance could be shown to some extent if the data from 2016 to 2017 was available. However, *S. pneumoniae* infections and PCV immunization are mainly in children under 5 years of age. This uneven distribution is consistent with the actual situation and enhanced the importance of this pathogen to younger children. Next, some important information, such as the subjects’ antibiotic use and vaccination experience, have not been collected, which could have contributed to an in-depth analysis of the current results. Finally, almost of the isolates were cultured from non-invasive specimens, sputum in particular (298/317), and only three strains were from invasive specimens. In the latest PCV position paper, the World Health Organization affirms that continuous or regular non-invasive isolate surveillance is helpful to understand the epidemiological characteristics of *S. pneumoniae (* World Health Organization, 2019).

In summary, the common serotypes of *S. pneumoniae* isolated from the children in Urumqi were types 19F, 19A, 23F, 6B, and 6A, in which there was no marked change found since the PCV13 introduction and the COVID-19 control. The serotype distribution of isolates from ethnic minority children was consistent with those from Han children in the same area. The coverage of PCV13 decreased since the vaccine was introduced into immunization, although this change was not statistically significant and some paradoxical phenomenon existed in specific PCV13 serotypes: 19A and 23F. The resistance rate to oral penicillin and ceftriaxone significantly declined in the COVID-19 control stage. Continuous survey will provide more perfect data for understanding the serotype distribution and drug resistance of *S. pneumoniae* in this region.

## Data availability statement

The raw data supporting the conclusions of this article will be made available by the authors, without undue reservation.

## Ethics statement

The studies involving human participants were reviewed and approved by Beijing Children’s Hospital ethics committee. Written informed consent from the participants’ legal guardian/next of kin was not required to participate in this study in accordance with the national legislation and the institutional requirements.

## Author contributions

X-HS, J-LT, JY, DW, JC, RA, and W-LZ collected the isolates in their local sites. K-HY and W-LZ applied the funds to support this work. M-YG, X-HS, K-HY, and W-LZ designed the study and interpreted the results. M-YG, WG, and LY confirmed the isolates and determined the serotypes of the isolates as well as the susceptibility test with E-test stripes. X-HS, J-LT, JY, DW, JC, and RA re-cultured the present isolates and determined the susceptibility with Compact system and KB discs. M-YG, X-HS, WG, and LY collected the data and performed statistics. M-YG, W-LZ, and K-HY draw the pictures and made the tables. M-YG and X-HS wrote the first draft of the manuscript. K-HY and W-LZ revised the manuscript according to all authors’ comments before submission. K-HY and W-LZ are responsible for the manuscript. All authors contributed to the article and approved the submitted version.

## References

[B1] Balsells E. Guillot L. Nair H. Kyaw M. H. (2017). Serotype distribution of streptococcus pneumoniae causing invasive disease in children in the post-PCV era: A systematic review and meta-analysis. PloS One 12 (5), e0177113. doi: 10.1371/journal.pone.0177113 28486544 PMC5423631

[B2] Ben-Shimol S. Givon-Lavi N. Greenberg D. van der Beek B. A. Leibovitz E. Dagan R. (2020). Substantial reduction of antibiotic-non-susceptible pneumococcal otitis media following PCV7/PCV13 sequential introduction. J. Antimicrob. Chemother. 75 (10), 3038–3045. doi: 10.1093/jac/dkaa263 32946586

[B3] China antimicrobial surveillance network(CHINET) (2021) The change of detection rate of MRSA, MRSE and other MRCNS from CHINET. Available at: http://www.chinets.com/Data/GermYear (Accessed January 3, 2023).

[B4] China National Clinical Research Center for Respiratory Diseases National Center for Children′s Health Group of Respiralogy Chinese Pediatric Society Chinese Medical Association Chinese Medical Doctor Association Committee on Respiralogy Pediatrics China Medicine Education Association Committee on Pediatrics. (2020). Chin. J. Appl. Clin. Pediatr. 35 (07), 485–505. doi: 10.3760/cma.j.cn101070-20200306-00329

[B5] Chinese Preventive Medicine Association Vaccine and Immunology Branch of the Chinese Preventive Medicine Association (2020). Zhonghua Liu Xing Bing Xue Za Zhi 41 (12), 1945–1979. doi: 10.3760/cma.j.cn112338-20201111-01322 33261246

[B6] Clinical and Laboratory Standards Institute (CLSI) (2019). “Performance standards for antimicrobial susceptibility testing,” in Twenty-ninth informational supplement(Wayne, PA). M100.

[B7] Lyu S. Hu H. L. Yang Y. H. Yao K. H. (2017). A systematic review about streptococcus pneumoniae serotype distribution in children in mainland of China before the PCV13 was licensed. Expert Rev. Vaccines 16 (10), 997–1006. doi: 10.1080/14760584.2017.1360771 28745918

[B8] Men W. Dong Q. Shi W. Yao K. (2020). Serotype distribution and antimicrobial resistance patterns of invasive pneumococcal disease isolates from children in mainland China-a systematic review. Braz. J. Microbiol. 51 (2), 665–672. doi: 10.1007/s42770-019-00198-9 31797324 PMC7203282

[B9] Moore M. R. Link-Gelles R. Schaffner W. Lynfield R. Lexau C. Bennett N. M.. (2015). Effect of use of 13-valent pneumococal conjugate vaccine in children on invasive pneumococcal disease in children and adults in the USA: analysis of multisite, population-based surveillance. Lancet Infect. Dis. 15 (3), 301–309. doi: 10.1016/S1473-3099(14)71081-3 25656600 PMC4876855

[B10] Platt H. Omole T. Cardona J. Fraser N. J. Mularski R. A. Andrews C.. (2022). Safety, tolerability, and immunogenicity of a 21-valent pneumococcal conjugate vaccine, V116, in healthy adults: phase 1/2, randomised, double-blind, active comparator-controlled, multicentre, US-based trial. Lancet Infect. Dis. doi: 10.1016/S1473-3099(22)00526-6 36116461

[B11] Satzke C. Turner P. Virolainen-Julkunen A. Adrian P. V. Antonio M. Hare K. M.. (2013). Standard method for detecting upper respiratory carriage of streptococcus pneumoniae: Updated recommendations from the world health organization pneumococcal carriage working group. Vaccine 32 (1), 165–179. doi: 10.1016/j.vaccine.2013.08.062 24331112

[B12] Sempere J. Llamosí M. López Ruiz B. Del Río I. Pérez-García C. Lago D.. (2022). Effect of pneumococcal conjugate vaccines and SARS-CoV-2 on antimicrobial resistance and the emergence of streptococcus pneumoniae serotypes with reduced susceptibility in Spain, 2004-20: A national surveillance study. Lancet Microbe 3 (10), e744–e752. doi: 10.1016/S2666-5247(22)00127-6 35932764 PMC9348823

[B13] van de Beek D. Cabellos C. Dzupova O. Esposito S. Klein M. Kloek A. T.. (2016). ESCMID guideline: diagnosis and treatment of acute bacterial meningitis. Clin. Microbiol. Infect. 22 Suppl 3, S37–S62. doi: 10.1016/j.cmi.2016.01.007 27062097

[B14] Wang J. Wu Q. S. Lu J. Ni Y. H. Zhou F. (2021). Low vaccination coverage of pneumococcal conjugate vaccines (PCVs) in shanghai, China: A database analysis based on birth cohorts from 2012 to 2020. Vaccine 39 (42), 6189–6194. doi: 10.1016/j.vaccine.2021.09.011 34538698

[B15] World Health Organization (2019) WHO position paper: Pneumococcal conjugate vaccines in infants and children under 5 years of age. Available at: https://www.who.int/publications/i/item/10665-310968 (Accessed November 20, 2022).

[B16] Zhou M. Wang Z. Zhang L. Kudinha T. An H. Qian C.. (2022). Serotype distribution, antimicrobial susceptibility, multilocus sequencing type and virulence of invasive streptococcus pneumoniae in China: A six-year multicenter study. Front. Microbiol. 12. doi: 10.3389/fmicb.2021.798750 PMC8793633 35095809

